# From phenotype to genotype in complex brain networks

**DOI:** 10.1038/srep19790

**Published:** 2016-01-22

**Authors:** Massimiliano Zanin, Marco Correia, Pedro A. C. Sousa, Jorge Cruz

**Affiliations:** 1 Science and Technology Faculty, Computer Science Department, Universidade Nova de Lisboa, Lisboa, Portugal; 2 Innaxis Foundation & Research Institute, José Ortega y Gasset 20, 28006, Madrid, Spain; 3 Departamento de Engenharia Electrotécnica, Faculdade de Ciências e Tecnologia, Universidade Nova de Lisboa, Lisboa, Portugal

## Abstract

Generative models are a popular instrument for illuminating the relationships between the hidden variables driving the growth of a complex network and its final topological characteristics, a process known as the “genotype to phenotype problem”. However, the definition of a complete methodology encompassing all stages of the analysis, and in particular the validation of the final model, is still an open problem. We here discuss a framework that allows to quantitatively optimise and validate each step of the model creation process. It is based on the execution of a classification task, and on estimating the additional precision provided by the modelled genotype. This encompasses the three main steps of the model creation, namely the selection of topological features, the optimisation of the parameters of the generative model, and the validation of the obtained results. We provide a minimum requirement for a generative model to be useful, prescribing the function mapping genotype to phenotype to be non-monotonic; and we further show how a previously published model does not fulfil such condition, casting doubts on its fitness for the study of neurological disorders. The generality of such framework guarantees its applicability beyond neuroscience, like the emergence of social or technological networks.

The “genotype to phenotype problem”, that is, the process of identifying the relationships between hidden variables (the genotype) and measured observables (the phenotype) of a system, has received increasing attention during the last decades. It originated in biomedicine as the process of mapping genes to their functions, a natural consequence of the ability of sequencing the full genome of a large number of different species^1^. More recently, it has been formulated within the study of complex networks. Initially, complex networks theory^2^,^3^,^4^ was used to analyse real-world networks, with the aim of extracting a set of topological features for understanding and controlling dynamical processes that take place over them. A paradigmatic example is the emergent phenomenon of synchronisation of networking oscillators, where the interplay between topology and dynamics has been largely clarified^5^. Networks were thus considered as independent entities, even though they are usually the result of evolutive processes, whose dynamic is defined by some parameters. In a way parallel to the genetic problem, the parameters of the evolutive process constitute a genotype space, while the resulting network properties are equivalent to the phenotype.

Numerous generative models have been proposed in the Literature. They started from theoretical models, *i.e*. models aiming at explaining one (or multiple) topological features independently on real-world networks. This category includes the achievement of scale-free degree distributions^2^,^6^,^7^, or the emergence of clustering structures^8^,^9^, modularity^10^,^11^, critical exponents^12^ and integration-segregation equilibria^13^. Such models have then been used to try to explain the emergence of numerous real-world networks, including metabolic^14^, transportation^15^,^16^,^17^, social^18^,^19^,^20^ or ecological ones^21^. A special attention has been devoted to brain networks: from cortical connectivity^22^,^23^,^24^,^25^, neural ensembles^26^,^27^, to fMRI^28^,^29^,^30^,^31^,^32^ and MEG^33^ functional networks.

In spite of this growing interest, some questions and doubts remain. When a new generative model is proposed, it ought to explain the reasons behind the appearance of some real-world network features, and thus to increase our understanding of the system under study. This usefulness has to be demonstrated, and thus there is a need for a *validation* framework. Additionally, two other aspects ought to be analysed: the criteria for the identification of the topological metrics to be modelled, thus of the elements of the phenotype that are of relevance; and the criteria for optimising the values of the hidden variables.

These three problems can be solved by leveraging on a classification task to validate the results of a genotype to phenotype mapping. Starting from a set of networks describing two (or more) different conditions (*e.g*. control subjects and patients suffering from some disease), the score obtained in the classification represents the quantity of information successfully revealed by the mapping. Such classification score can then be used as a criterion for the optimisation and validation of the generative model. We apply the proposed validation framework to the study of functional brain network, and specifically to the *Economical Clustering* model presented in refs 30,32, to show that it does not yield additional information about the mechanisms governing the appearance of neurological disorders. We further provide a minimal requirement for obtaining relevant genotype to phenotype mappings, *i.e*. the non-monotonicity of the relation between hidden variables and network topological metrics.

## Selection of topological metrics

If one is to construct a model mimicking the growth of a functional brain network, or in general of any real-world network, the first question to be addressed is which topological metrics should be modelled. Any real network can be characterised by tens (if not hundreds) of topological metrics, which have been developed in the literature during the last decade^34^. It is nevertheless not advisable to model all topological features: many of them may be redundant (*i.e*. already codified by a similar metric), or not relevant for the process under study. It is thus customary to select a few of them, expected to be relevant according to some expert judgement criteria, and construct a model able to recover such properties. It is also important to notice that most of them are defined for unweighted networks, while the standard output of a functional analysis is a fully-connected weighted graph. The researcher then ought to binaries such graph, by applying a threshold that should be chosen according to some criteria.

Numerous brain network topological features have been studied through network models: link density^24^, modularity^22^,^23^,^30^, hub structures and rich club^23^,^32^, clustering coefficient^22^,^30^, or outreach^33^. The choice of the topological metrics and thresholds to be studied has mainly been left to the experience of the researcher, a procedure that does not guarantee the optimality of results. A more pragmatic solution has been proposed in ref. 35. Given two or more groups of individuals, as for instance control subjects and people suffering from some diseases, the metrics to be studied are those that maximise the score in a classification task, *i.e*. a task designed to correctly classify subjects in those groups (see Methods for a discussion of the classification process). The rationale behind this is that the higher the classification score obtained, the more information about the structural differences between the two groups is encoded by the metric(s) considered. This thus provides an objective criterion for metrics and threshold selection.

**Figure 1:**
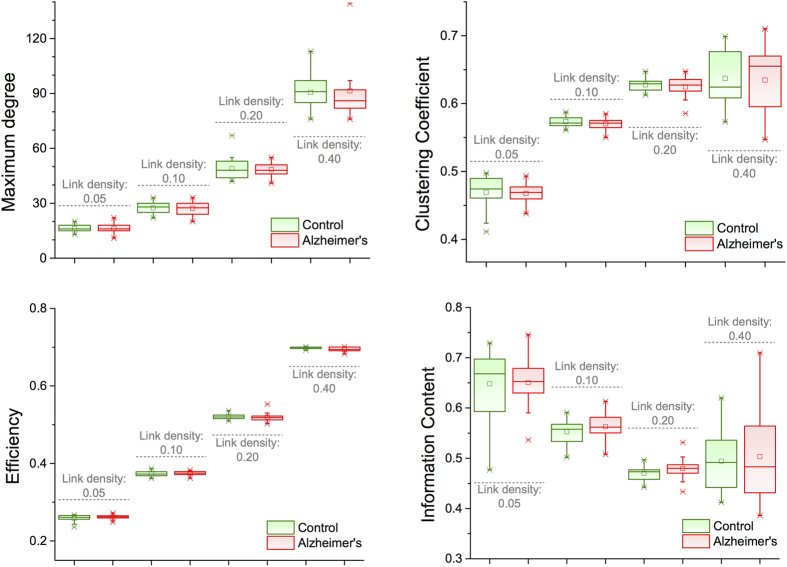
Topological metrics evolution for control and Alzheimer’s subjects. Each panel reports the box plots corresponding to the distribution of a topological metric in control subjects (green) and Alzheimer’s patients (red). Four link densities are considered: from left to right inside each panel, 0.05, 0.1, 0.2 and 0.4. From left to right, top to bottom, the four topological metrics reported are the maximum degree, clustering coefficient, efficiency and Information Content (see Methods for definitions).

It is further important to understand how topological metrics and thresholds interact, usually in a non-trivial way. Figure 1 reports the evolution of the probability distributions associated to four topological metrics (maximum degree, clustering coefficient, efficiency and Information Content, see Methods for details) and four link densities (0.05, 0.1, 0.2 and 0.4), for two groups of control subjects and Alzheimer’s patients - see Methods for details on the network reconstruction technique. In most cases, the two distributions are similar; thus, for instance, studying the clustering coefficient for a link density between 0.05 and 0.2 would not yield any information about the pathology under study. More than one threshold may also ought to be considered at the same time; for instance, the maximum degree and the Information Content can be both informative about the pathology, but at different link densities: 0.4 for the former, 0.1 for the latter.

The score obtained in a classification task thus provide a first criterion for the selection of the topological metrics to be included in the generative models, and of the corresponding thresholds: only those combinations that allows discriminating between the groups of subjects ought to be considered^35^. While this is generally true, there exists situations in which metrics yielding low statistical significance may have a value, providing the phenotype to genotype transformation is non-monotonic and able to amplify little differences between the two groups. An example of this process will be developed in Section.

## Model parameters optimisation

At this stage, the researcher should have defined two important aspects. First, the set of metrics describing the real networks under analysis, *i.e*. the phenotype. Each one of these metrics is described by a probability distribution, as obtained from the set of real networks. Second, a network growth model, to be tested and validated. Given a topological metric *m*, two probability distributions are available: the one obtained from real data 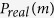, and the one resulting from the model 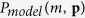, being **p** the set of parameters governing the model properties. Moving from phenotype to genotype thus requires defining the optimal value of **p**, p^*^, such that 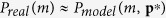.

The usual method involves, for each possible set of parameters **p**, creating a large number of networks, extract their topological characteristics, and then compare the obtained distributions with the real one. Several methods have then be used to compare and optimise such distributions. The simplest solution is, of course, to perform a graphical analysis of the results^29^,^33^: while extremely intuitive for the reader, such an analysis does not guarantee the optimality of results, nor their statistical significance.

A more sophisticated solution involves the calculation of the *p*-values corresponding to some statistical test; while this may guarantee the statistical significance of the fit, it still has some important problems. First, obtaining a *p*-values usually implies defining the probability distribution associated to the data, which, in many real cases, may not be a standard one, *e.g*. Gaussian, and this hypothesis should be tested. The whole shape of the distribution should be tested, as clearly two distribution may share the average, but yet be different in other momenta. Additionally, the computational cost associated to such analysis may be significant. Several hundreds of networks ought to be constructed and analysed for each set of parameters **p**. Finally, the number of **p**s to be analysed is usually exponential with the number of parameters considered. Even with just two or three parameters, the final number of networks can easily be of the order of the million.

Refs 30,32 proposed a further improvement to the *p*-value calculation, by using an energy function of the form:


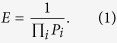



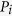
 here represents the *p*-value of the Kolmogorov-Smirnoff (K-S) test between the distributions estimated from the simulated and experimental networks, and *i* runs over all topological metrics. Such solution solves two problems at the same time: by resorting to the K-S test, the shapes of the distributions are not longer a problem, providing enough data are available to describe the empirical ones; and it allows to optimise several parameters at the same time, thus finding an optimal compromise between them.

**Figure 2:**
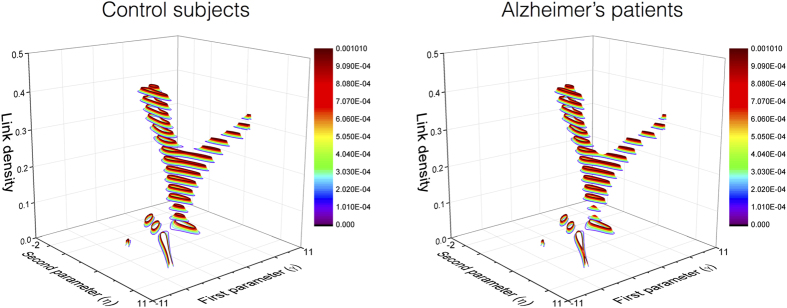
Parameters space obtained through the PCP method. Left and right panels respectively represent control subjects and AD patients. The colour of each contour line represents the normalised probability of generating networks topologically equivalent to the real ones, as a function of the link density and of the two parameters of Eq. 2. See main text and ref. 36 for further details on the PCP method.

Finally, all the previously described techniques are characterised by an hidden assumption: the aim of the analysis is to obtain the best set of parameters, recovering the structure of the experimental networks. It may nevertheless be important to also characterise the region of space in which those parameters can be accepted, for a given level of statistical significance. In other words, it may be relevant to move from a point, to a space region. This would allow, for instance, to estimate the sensitivity of the model to parameter changes; and to assess the presence of multiple local minima, which, although not optimal, may be of biological relevance. The use of Probabilistic Constraint Programming (PCP) has been proposed as a way of fully reconstruct the parameters space associated to a generative model^36^. PCP extends the classical Constraint Satisfaction Problem framework with a Monte Carlo approach to estimate the probability associated to each region of the space. Figure 2 depicts the probability distribution obtained by the Economical Clustering model (see next Section and Eq. 2), for several link densities, both in healthy and AD patients. Two features need to be noticed. First, for high link densities, two parameters regions yield networks with characteristics that are statistically similar to the one obtained from the real data, thus suggesting that minimising a *p*-value is not enough to have a complete picture of the problem. Second, the probability distributions for both groups of subjects are extremely similar: as discussed in the next Section, this has important implications in terms of the model validation.

## Model validation

Once the topological features to be modelled have been selected, and the parameters of the explanatory model have been optimised, one last question is left: is the model really explaining the networks we see in the real world? In other words, does the model yield additional information about the system under study? It is worth stressing the importance of this question. If no additional knowledge is yielded, then the modelling process will remain a nice mathematical speculation. The final aim of any neuroscience analysis is, nevertheless, to provide new knowledge about brain functioning and its pathological states, thus improving our diagnostic and prognostic capabilities.

Taking into account this aim, one can follow the methodology presented in ref. 35, and adapt it to the phenotype to genotype problem: given a classification problem, *e.g*. discriminating between healthy subjects and patients, the genotype (the model parameters) should improve the classification score with respect to the phenotype (topological metrics) alone. By denoting the topological metrics in the real networks by 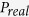, and the best model parameters by p^*^ (as fitted in the previous step), the score obtained by a model trained using p^*^ should be higher than the score obtained by using 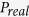. If this does not occur, then p^*^ are just a transformation of 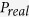 that does not encode any additional information.

It is always possible to define a function mapping the genotype to the phenotype, *i.e*. 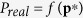, as this function is the result of transforming the genotype to a network, and the latter into a set of topological features. *f* is thus defined by the model used, by its parameters, and by the network metrics considered. The problem of creating a relevant generative model is thus equivalent to defining an *f* that makes accessible information that may be encoded in 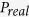, but that is not readily accessible to a classification algorithm.

While it is not simple to define a criterium to assess whether this condition is fulfilled, it is nevertheless straightforward to define several *f*s that do not provide additional information.

The simplest case is a linear transformation, such that 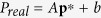, *A* and *b* being respectively a real matrix and vector. Here, the transformation genotype-phenotype is simply an affine transformation of the space created by the model parameters, which is not relevant for any robust classification algorithm. Notice that the only exception are Decision Trees, although the optimal classification can be recovered by applying a Principal Component Analysis prior to the classification, as in the Rotation Forest algorithm^37^. A similar result should be expected when *f* is a monotonic function, as the relative position of control subjects and patients in the genotype space is conserved in the phenotype one.

**Figure 3:**
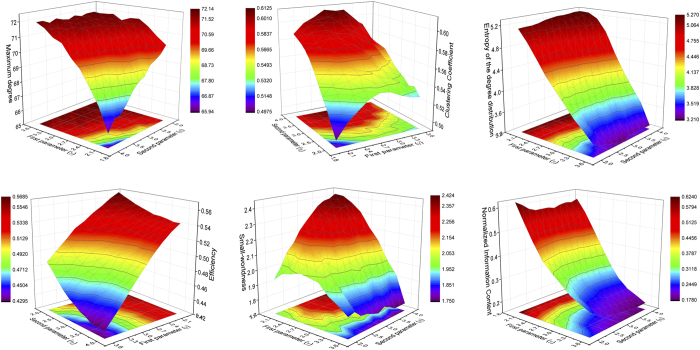
Topological metrics evolution in the Economical Clustering model. Each panel reports the evolution of a topological metric as a function of the two model parameters, *γ* and *η* - see Eq. 2. From left to right, top to bottom, the six considered metrics are the maximum degree, clustering coefficient, entropy of the degree distribution, efficiency, small-worldness and Information Content - see Methods for definitions.

Most generative models used in the literature yield monotonic *f*s, which in many cases are just polynomial functions. As an example, one may consider the Economical Clustering model presented in refs 30,32. The probability of connecting two nodes *i* and *j* is defined as:


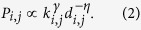



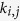
 is the number of neighbours common to *i* and *j*, and 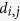 is the physical distance between the two nodes. *γ* therefore controls the appearance of triangles in the network (*i.e*. its clustering coefficient), and 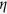 accounts for a distance cost in the connections. Using information about sensors distance in a MEG machine, it is possible to recover the evolution of some topological metrics as a function of the two parameters - see Fig. 3. Results indicate that all six metrics considered in Fig. 3 are a monotonic transformation of the two phenotype parameters, and that some of them (the entropy of the degree distribution and the Information Content) can be well approximated by a linear transformation.

In order to corroborate the hypothesis that the monotonic transformation associated to Eq. 2 cannot increase the yielded information, the classification error obtained by third-order polynomial transformations, both monotonic and non-monotonic, have been assessed. Specifically, the clustering coefficient and Information Content metrics for control subjects and Alzheimer’s patients networks, are transformed according to:


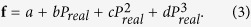


The coefficients *a*, *b*, *c* and *d* are randomly drawn from an uniform distribution 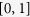, and discarded whenever the function 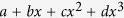 is not monotonic for 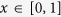. **f** thus emulates the phenotype parameters that may be obtained by fitting a large family of network generative models, in which the functions relating **f** and 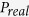 are monotonic. **f**s are further manipulated by introducing a *spread* between control subjects and patients:


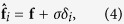



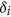
 being 1 if the subject *i* is a control, −1 otherwise. The larger *σ*, therefore, the larger the separation between both groups, and thus the lower the expected classification error. A Decision Tree classification model is then trained using the features 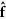, and the error in the classification assessed through a Leave One Out validation^38^ (see Methods for details).

**Figure 4:**
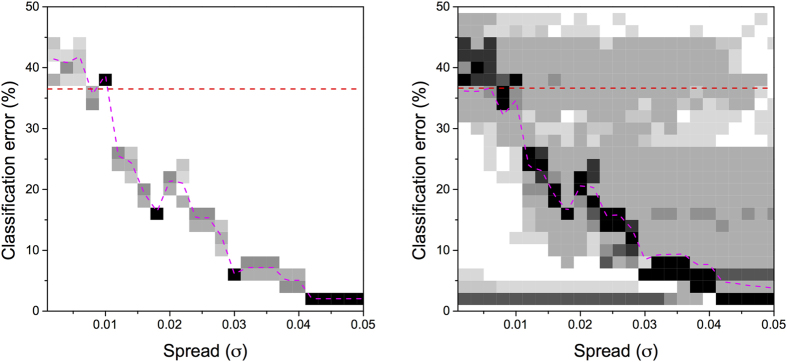
Probability distributions of classification errors. Left and right panels represent the distribution of the probability of finding a classification error, when a monotone (top) and non-monotone (bottom) transformation are used. Black and light grey squares respectively indicate maximum and minimum probabilities. The pink dashed lines indicate the average error, while the horizontal red ones the classification error obtained from the original features 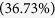. Results are reported as a function of the *spread σ*, as defined in Eq. 4.

This allows us to compare three classification problems. First, the classification using the original features, such that the class (healthy or suffering from Alzheimer’s) of each subject is predicted using the corresponding values of the clustering coefficient and Information Content (as encoded in 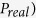. Second, the classification using the monotonically transformed topological features, in which the class is predicted according to the values obtained through Eqs 3 and 4. Third, the same classification process, but considering non-monotonic transformations in Eq. 3. Figure 4 Left reports the distribution of classification errors as a function of *σ* for the second classification problem, Fig. 4 Right for the third. It can be seen that, in the former case, classification errors are clustered together, while in the latter one they extend vertically above and below the average value. In other words, a monotonic transformation cannot improve the results that would be obtained with the original features, in this case the topological metrics extracted from the real networks. On the contrary, a non-monotonic transformation is able, in some cases, to introduce enough variability to improve the classification process.

It should be noticed that an increase in the classification score is not, by itself, a sufficient condition, but only a necessary one. Specifically, the incremented score can be just the result of random perturbations in the data, an effect known as *curse of dimensionality*^39^. The researcher must thus confirm that two conditions are fulfilled: that the classification score is improved, and that the generative model has a sound biological foundation.

## Discussion

Three common pitfalls undermine the use of generative models to understand the growth of functional networks. First of all, generative models are usually used to explain the appearance of some specific topological properties, whose choice is not driven by objective criteria. Due to the way networks are analysed, those properties are defined for a given link density in unweighted graphs. It is then important to carefully choose such metrics and link densities, to ensure that they are really representative of the task or pathological condition under analysis. Second, the process of obtaining the best model parameters describing the real networks is not a straightforward process, as it requires the adoption of some statistical criteria. Lastly, even if the model is able to recover some topological characteristics, this does not guarantee that the generative model itself is representative of what is really happening in the brain, *i.e*. that the model yields new knowledge of the neurological processes developing during a task.

Such problems can be tackled by means of a classification task, in which two conditions (*e.g*. control subjects, and people suffering from some disease) are compared. An increase in the knowledge about the conditions implies a higher capability of discriminating between both states; this, in turn, implies that the generative model should increase our capacity of discriminating between both conditions, in order to ensure an increase in knowledge.

The result is a framework composed of three processes:

1. Identification of the topological metrics to be modelled, and of the corresponding thresholds, by selecting those that are relevant in a classification task, and that therefore encode useful information.
2. Creation of a suitable generative model. The relationship between the phenospace (*i.e*. the observed topological metrics) and the genospace (the model parameters) should at least be a non-monotonic function. If this condition is not fulfilled, the use of a generative model is equivalent to a transformation of the phenotype space that maintains the relative position of both groups of subjects, and that thus provide no additional information.
3. Validation of the whole model, by assessing the increase in knowledge (decrease in the classification error) it yields.

We suggest that such framework should be the basis of any network analysis based on generative models; not only of brain functional networks, but also of transportation, social or ecological ones.

## Methods

### Data set and functional networks reconstruction

Forty-nine right handed elderly participants recruited from the Geriatric Unit of the Hospital Universitario San Carlos Madrid and the Centro de PrevenciÃ^3^n del Deterioro Cognitivo, Ayuntamiento de Madrid, participated in the study. Participants were divided into two groups according to their clinical profiles: 30 participants were considered as Alzheimer’s patients, and 19 as elderly control participants. The average number of years of education in both groups was similar, i.e. 10 years for patients and 11 years for controls. Before the task execution, all participants or legal representatives gave informed consent to participate in the study. The study was approved by the ethics committee of the Hospital Universitario San Carlos Madrid, and has been performed in accordance with relevant guidelines and regulations.

Magneto-encephalographic (MEG) scans were obtained in the context of a modified version of the Sternberg’s letter-probe task^40^ in which a set of five letters was presented and participants were asked to keep the letters in mind. After the presentation of the five-letter set, a series of single letters (500 ms in duration with a random ISI between 2 and 3 s) was introduced one at a time, and participants were asked to press a button with their right hand when a member of the previous set was detected. Participants undertook a training series, and the actual test only started when participants demonstrated that he/she remembered the five-letter set.

The MEG signal was recorded with a 254 Hz sampling rate, and a band pass filter between 0.5 to 50 Hz; the recording was performed using 148-channel whole head magnetometer, confined in a magnetically shielded room (MSR). An environmental noise reduction algorithm using reference channels at a distance from the MEG sensors was applied to the data, and single trial epochs where visually inspected by an experienced investigator and epochs containing visible blinks, eye movements or muscular artefacts were excluded from further analysis. Artefact-free epochs from each channel were then classified into four different categories according to the subjects performance in the experiments: hits, false alarms, correct rejections and omissions, of which only hits were considered for further analysis. 35 1 second-long epochs were randomly chosen from those corresponding to correct answers for each of participant. A synchronisation matrix 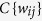 of size 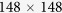 was then computed for each participant from the MEG time series, by means of the Synchronization Likelihood (SL) algorithm^41^.

### Networks topological analysis

Each synchronisation matrix 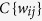 has been binarised, by conserving only those links above a given threshold (see main text for a discussion of the selection criteria), and then analysed using a set of standard topological metrics.

- Maximum degree. Maximum number of links departing and arriving from a node.
- Efficiency^42^. This metric was proposed to overcome the limitation of the mean geodesic path length, which diverges when the network is disconnected. It is defined as the harmonic mean of the distances between pairs of nodes.
- Clustering coefficient^43^. The clustering coefficient, also known as transitivity, measures the presence of triangles in the network, and is defined as the relationship between the number of triangles in the network and the number of connected triples.
- Entropy of the degree distribution^44^. Defined as the Shannon entropy of the nodes degree distribution, it provides a measure of the heterogeneity of the network: the maximum value is obtained for a uniform degree distribution, while the minimum is achieved whenever all vertices have the same degree.
- Small-worldness^45^,^46^. Ratio between the clustering coefficient and the mean geodesic path length of a network, normalised by the mean value obtained in comparable random graphs.
- Information Content^47^. Metric assessing the quantity of information needed to reconstruct the network by starting from a single node. It thus assesses the presence of meso-scale regularities in the adjacency matrix, as for instance community structures.

### Classification task

In general terms, a classification task, also known as *supervised learning*, tries to learn from a set of training data, with the final aim of predicting the class of new unlabelled records^38^. More specifically, let *X* be the feature space and its possible values, *i.e*. a set of *observables* describing all records; and *Y* be the space of possible labels, *e.g*. healthy or suffering from some disease. The underlying assumption is that it exists a function 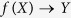 that assigns a record to a class depending on the values of its describing features. A classification task thus entails identifying (or approximating) the function *f*, in order to be able to label new records. Notice that the number of labels is not limited to two, as for instance when the analysed subjects may suffer from multiple diseases, provided that the classification algorithm is suited to handle such scenario.

In this paper we use *Decision Trees* (DT) as a prototypical example of a classification algorithm^48^,^49^. DT aims at generating comprehensible tree structures that classify records by sorting them based on attribute values. Each node in a decision tree represents an attribute in a record to be classified, and each branch represents a value that the attribute can take. Once the tree has been created, features of new non-classified records are compared against the structure to predict the corresponding labels. The DT implementation of the KNIME software^50^ has here been used.

The accuracy of the classification model is usually evaluated through its *score*, *i.e*. the proportion of times the model is able to accurately predict the class of a record. In order to avoid overfitting, it is necessary to evaluate this score on records that are not part of the training data set - *i.e*. to obtain a *generalisation error*, or the measure of the performance of the model in a real environment. We here estimate the classification score using a *Leave-One-Out Cross Validation* (LOOCV)^38^,^51^, the standard choice when the number of available records is limited. A single observation from the original sample is used as validation data, and its class is predicted by training the model using all other records. This process is repeated for all records available, and the global score is obtained as the average classification success rate.

## Additional Information

**How to cite this article**: Zanin, M. *et al*. From phenotype to genotype in complex brain networks. *Sci. Rep*. **6**, 19790; doi: 10.1038/srep19790 (2016).
